# Evaluation of the secondary use of electronic health records to detect seasonal, holiday-related, and rare events related to traumatic injury and poisoning

**DOI:** 10.1186/s12889-020-8153-7

**Published:** 2020-01-13

**Authors:** Timothy Bergquist, Vikas Pejaver, Noah Hammarlund, Sean D. Mooney, Stephen J. Mooney

**Affiliations:** 1 Department of Biomedical Informatics and Medical Education, University of Washington, Box 358047, Seattle, Washington 98195-8047 USA; 2 Department of Epidemiology, University of Washington, Seattle, WA USA; 3 Harborview Injury Prevention & Research Center, University of Washington, Seattle, WA USA

**Keywords:** Learning healthcare system, Data science, Population health, Electronic health records

## Abstract

**Background:**

The increasing adoption of electronic health record (EHR) systems enables automated, large scale, and meaningful analysis of regional population health. We explored how EHR systems could inform surveillance of trauma-related emergency department visits arising from seasonal, holiday-related, and rare environmental events.

**Methods:**

We analyzed temporal variation in diagnosis codes over 24 years of trauma visit data at the three hospitals in the University of Washington Medicine system in Seattle, Washington, USA. We identified seasons and days in which specific codes and categories of codes were statistically enriched, meaning that a significantly greater than average proportion of trauma visits included a given diagnosis code during that time period.

**Results:**

We confirmed known seasonal patterns in emergency department visits for trauma. As expected, cold weather-related incidents (e.g. frostbite, snowboarding injury) were enriched in the winter, whereas fair weather-related incidents (e.g. bug bites, boating accidents, bicycle accidents) were enriched in the spring and summer. Our analysis of specific days of the year found that holidays were enriched for alcohol poisoning, assaults, and firework accidents. We also detected one time regional events such as the 2001 Nisqually earthquake and the 2006 Hanukkah Eve Windstorm.

**Conclusions:**

Though EHR systems were developed to prioritize operational rather than analytic priorities and have consequent limitations for surveillance, our EHR enrichment analysis nonetheless re-identified expected temporal population health patterns. EHRs are potentially a valuable source of information to inform public health policy, both in retrospective analysis and in a surveillance capacity.

## Background

### Electronic health records and meaningful use

The past decade has seen a substantial increase in the rate of Electronic Health Record (EHR) adoption in healthcare [1]. While the primary drivers of EHR adoption have been the 2009 HITECH act and the data exchange capabilities of EHRs, [2] secondary use of EHR data to improve patient safety and health is a key benefit of large-scale adoption [3]. EHRs contain a rich set of information about patients and their health experiences, including doctor’s notes, medications prescribed, and billing codes [4]. As hospitals improve data capture quality and quantity, opportunities arise for meaningful use of the data outside the clinic.

### Electronic health records and public health

Public health surveillance -- monitoring disease prevalence, and the conditions and behaviors that affect prevalence -- is a core component of preventive medicine. Surveillance is conventionally categorized as either ‘active’ (wherein a health authority contacts care providers or the public to assess conditions) or ‘passive’ (wherein care providers are mandated to report certain conditions to the health authority) [5]. For example, the Center for Disease Control’s Behavior Risk Factor Surveillance System (BRFSS), [6] in which trained interviewers contact tens of thousands of respondents by phone each year, is an active system. By contrast, the National Highway Transport Safety Administration’s Fatality Analysis Reporting System, in which state transportation departments report motor vehicle crashes to a central system, is a passive system.

With the increasing adoption of EHRs, automated and scalable public health surveillance has become possible. Clinical data that is collected in routine medical care can be algorithmically processed for syndromic surveillance, a passive reporting technique wherein patient cases of a particular disease or condition relevant to population health (frequently, but not exclusively infectious disease) are automatically flagged and reported to appropriate authorities in real time. EHRs have been shown to be a reliable data source capable of facilitating syndromic surveillance [7–11]. The prevalence estimation of EHRs have also been shown to accurately reflect the known prevalence of a served region. For example, when compared to the gold standard BRFSS dataset, Klompas et al. found that an EHR-based diabetes prevalence detection algorithm was nearly as accurate as the BRFSS dataset [8]. Perlman et al. found that measures of smoking prevalence, obesity rates, hypertension, and diabetes that were derived from the EHR were as accurate as the gold standard BRFSS datasets [12]. The reliability of different conditions often differs by healthcare system, but as more sites adopt EHRs, the estimates should improve for more conditions [13].

Previous efforts to use EHRs for public health reporting have revolved around using syndromic surveillance to electronically report cases to a data repository external to the EHR. For instance, Klompas et al. developed a platform for integrating EHR data for use in public health called the Electronic medical record Support for Public Health (ESP) [14]. The platform enabled automated systems to pull relevant records from the EHR, and then aggregate data for visualization and analysis in an application called RiskScape [7]. A more recent example of integrating clinical data into a repository for public health surveillance was the Public Health Community Platform (PHCP), an attempt by multiple public health organizations (APHL, ASTHO, JPHIT) to standardize and develop a platform for EHR to cloud-based public health data sharing and electronic case reporting [14, 15]. While the pilot study faced several challenges, it demonstrated long-term feasibility for widespread integration between clinical practice and public health.

### The EHR as a generalizable population health surveillance platform

While syndromic surveillance typically focuses on the detection and prevalence estimation of specific conditions, electronic health record databases can act as a generalized population health surveillance system, giving insight into previously unmonitored diseases. For instance, Melamed et al. showed the utility of EHRs to link diseases to seasonal trends [16]. Other seasonal detection methods using EHR data have been used to model seasonal influenza outbreaks, seasonal blood pressure controls, and seasonal effects on early child development [17–19]. While these studies show that EHRs can be used for accurate population health trends, each of these have looked at only one category of disease at a time.

In this paper, we explore the utility of the EHR as a generalizable event and trend detection platform. In contrast to previous studies, we don’t look for seasonal trends of specific diseases, but rather look for unusual coding trends for all traumatic injuries because they have known seasonal trends [16–18] and gold standard events by which we can validate a generalizable event detection method (e.g., we expect the 4th of July to have a spike in firework accidents). Our goal is to test whether a general event detection method can use a live EHR system to alert public health officials to possible actionable environmental events. We look at deviations from seasonal and temporal trends in medical information collected in routine clinical care, conceptualizing these deviations as events of potential interest to authorities tasked with monitoring population health. We externally validate flagged code/time period combinations, confirming that a holiday or rare event was likely the cause of the unusual injury pattern.

Throughout this paper, we use the term “detection” to refer to the association of statistical trauma trends with individual dates or seasons (e.g., can we “detect” winter or July 4th based on relative diagnosis code frequencies?). We look for diagnosis codes that are statistically “enriched” (a greater proportion of overall visits than would be expected due to chance alone) for different periods of time. We define a code as “enriched” when that code is significantly associated with a given period of time [20]. For instance, we expect injuries from snow sports like skiing, snowboarding, and snowmobiling to be “enriched” in the winter months. We compare trends found to expected trends from literature and common knowledge to test the validity of this event detection technique.

## Methods

### Data source

We obtained a data set (diagnoses by date) from the UW Medicine (the University of Washington Health System) enterprise data warehouse (EDW). The EDW includes patient data from over 4.5 million patients spanning ~ 25 years, and representing various clinical sites across the UW Medicine system including University of Washington Medical Center, Harborview Medical Center, and Northwest Hospital and Medical Center.

“Injury and poisoning” is a category of clinical affliction that includes any traumatic injury or poisoning and is coded as E-codes (E000-E999) or 800–999 codes using the ICD-9-CM diagnosis coding standard or S00-T99 or V00-Y99 codes using the ICD-10-CM coding standard, as defined in the CDC’s guidelines for traumatic injury and poisoning [21, 22]. From the EDW, we selected records of all visits between January 1, 1994 and May 2, 2017 for patients who were over the age of 18 as of May 2, 2017 and where, for each visit, at least one ICD-9-CM code or ICD-10-CM code in the “Injury and poisoning” category was recorded. For each patient record, we collected patient visit information which included de-identified patient ID, diagnosis coding method (ICD-9-CM or ICD-10-CM), visit number identifier, admission date and time, diagnosis codes (ICD-9-CM or ICD-10-CM), and diagnosis code description. These data represent just over 3,000,000 unique trauma-related visits to the UW medical system made by over 650,000 unique individuals.

### Data cleaning

UW Medicine adopted the ICD-10-CM billing code system in mid-2015. In order to ensure we had consistent data throughout, we mapped ICD-10-CM codes to their ICD-9-CM equivalents, using the Center for Medicare and Medicaid Services (CMS) General Equivalence Mappings [23]. Since ICD-10-CM has more detailed coding descriptions than ICD-9-CM, there is a potential for data loss when converting from ICD-10-CM to ICD-9-CM. While this may be an issue in some studies, we were more interested in the high level view of UW’s patient population, and this data loss was not a major concern for this study. We used a custom tool, DxCodeHandler (https://github.com/UWMooneyLab/DxCodeHandler), to handle code conversion, ICD hierarchy traversal, and diagnosis code manipulation (Additional file 1).

### Obtaining count data

Per our selection criteria, each patient visit included one or more ICD-9-CM or ICD-10-CM billing codes representing the billing information for the patient visit. We attributed all codes appearing in a visit to the day that visit occurred such that each day was considered a collection of independent code counts. We also included all higher level categories in the ICD hierarchy along with the low level codes. For example, a day that had the code E880.0 (Accidental Fall on or from Escalator) would also have E880 (Accidental Fall from Stairs or Steps), E880-E888 (Accidental Falls), and E000-E999 (External Causes of Injury or Poisoning) counted on that day. This incorporation of multiple category levels was necessary because some real world events enrich different classes of injury such as large classes of injury (e.g. 800–829, Fractures), mid-level classes of injury (e.g. 989, Toxic Effect of Non-medicinal Substances), or specific injury types (e.g. 854.06, Intracranial injury with loss of consciousness).

### Binomial test and hypothesis testing

For each diagnosis code, both billable and parent codes, we tested the null hypothesis that the prevalence of each diagnosis code, when calculated against all trauma visits, was consistent across time. We tested this hypothesis using a binomial test, where we tested whether a diagnosis code is more or less prevalent in a given time period when compared to the expected prevalence if the null hypothesis were true. If a code-time period pair had a *p*-value less than the Bonferroni cutoff, we said that the code is enriched for that tested time period. We used an ɑ = 0.01 when calculating the Bonferroni cut off for each experiment. We ran this test for every code that appears more than 10 times in our dataset for all four seasons and for all 365 (non-leap year) days. For each code-time period pair, we generated a score by calculating the -log(p-value) from the binomial test.

### Enrichment of seasons

To find seasonal statistical enrichment of ICD-9-CM billing codes we summed daily counts of each of the 4582 poisoning and injury billing codes within each season. We defined Winter as December–February, Spring as March–May, Summer as June–August, and Autumn as September–November. For each season/code pair, we performed a binomial test, treating the sum of all codes in that season as the trials, and the count of the code in question for that season as the successes. The expected rate of appearance for each code in question was established by calculating its proportion of all trauma visits across all seasons and years. Thus, the *p*-value from this test is interpretable as the probability that these many codes or more would be seen in a given season under the null hypothesis that codes are evenly distributed across the year. We used a Bonferroni correction at *n* = 18,328 (4 × 4582). We also filtered out codes that appeared less than 10 times over the course of the 24-year period.

### Enrichment of dates

We used an analogous method to detect code enrichments for days of the year. Again, we computed the sum of codes occurring on each of the 365 (non-leap-day) days of the year. For each code/day pair, we performed a binomial test using the total number of codes used on that day as the number of trials, and the number of times the specific code of interest was used as the number of successes. The expected rate was derived from the baseline rate of appearance for the code of interest per day across the entire year when compared to the total number of trauma visits on that given day. We calculated a Bonferroni cutoff at n = 1,672,430 (4582 × 365). We counted codes as enriched if the p-value was less that the Bonferroni correction and the daily rate of the code was greater than the baseline expected rate of the code (we did not look at depletions). We also filtered out codes that appeared less than 10 times over the course of the 24 year dataset period.

### IRB considerations

We received an IRB non-human subjects research designation from the University of Washington Human Subjects Research Division to construct a dataset derived from all patient diagnoses from the EDW over the age of 18. (IRB number: STUDY00000669) Data was extracted by an honest broker, the UW Medicine Research IT data services team, and no patient identifiers were available to the research team.

## Results

### Statistical enrichment of seasons

We detected patterns of seasonal enrichment consistent with our expectations about seasonal behavior. For example, in winter, we found enrichment of not only accidents from snow sports such as skiing and snowboarding, among others, but also cold weather-related ailments such as frostbite and hypothermia. Other codes that may be related to snow sport accidents such as head injuries, sprains, and strains were also enriched (Table 1). Spring begins to have more fair weather activities such as outdoor related ailments like allergies and sporting accidents (Table 2). Summer sees disproportionate numbers of accidents related to outdoor activities in warm weather such as bites and stings from bugs, firework accidents, bicycle accidents, and water transport accidents (Table 3). While fall is the least distinctive of the seasons, it has a unique enrichment for vehicle accidents (Table 4). This may be because fall contains high traffic holidays (Thanksgiving, Labor Day) and increased levels of rain in Seattle.

**Table 1:** Top 20 most enriched codes for Winter. The top 20 most enriched codes for Winter. Enriched codes include accidents from snow sports such as skiing and snowboarding as well as cold weather-related ailments such as frostbite and hypothermia. Other codes that may be related to snow sport accidents such as head injuries, sprains, and strains were also enriched. We report by percent increase as well as -log(p). We compare the number of codes found in Winter to the average code counts of the other three seasons

ICD 9 Code	Description	Winter Code Counts	Average Counts in Other Seasons	Percent Increase	*P* Value	Scores
E885.4	Fall From Snowboard	831	115	622.61	0.00	750.00
E885.3	Fall From Skis	593	122	386.07	2.59E-221	507.92
991	Effects of Reduced Temperature	2027	995.33	103.65	5.16E-217	498.02
E885	Fall on Same Level From Slipping, Tripping, or Stumbling	10,738	9019	19.06	6.67E-140	320.46
996–999	Complications of Surgical and Medical Care	134,022	135,432	−1.04	3.24E-137	314.28
995.29	Unspecified Adverse Effect of Other Drug, Medicinal and Biological Substance	5019	3751.33	33.79	1.61E-134	308.07
996	Complications Peculiar to Certain Specified Procedures	88,630	88,559	0.08	4.72E-122	279.36
E930-E949	Adverse Effects From Substance in Therapeutic use	16,422	15,087.67	8.84	1.60E-92	211.37
E820	Nontraffic Accident Involving Motor-driven Snow Vehicle	239	51.33	365.61	7.72E-87	198.28
995.2	Other and Unspecified Adverse Effect of Drug, Medicinal and Biological Substance (due) to Correct Medicinal Substance Properly Administered	8648	7517.33	15.04	7.82E-86	195.97
991.2	Frostbite of Foot	372	118.67	213.47	1.25E-85	195.5
E003.2	Activities Involving Snow (alpine) (downhill) Skiing, Snow Boarding, Sledding, Tobogganing and Snow Tubing	148	19.67	652.41	1.50E-80	183.8
E901.0	Accident due to Excessive Cold due to Weather Conditions	334	104.67	219.1	6.42E-79	180.04
E003	Activities Involving Snow and ice	169	27.67	510.77	1.81E-78	179.01
E901	Excessive Cold	474	201.33	135.43	1.25E-69	158.65
E880-E888	Accidental Falls	38,739	38,299.67	1.15	1.37E-68	156.26
991.6	Hypothermia	760	414	83.57	1.01E-64	147.36
E885.9	Fall From Other Slipping, Tripping, or Stumbling	8953	8067.67	10.97	3.03E-63	143.95
990–995	Other and Unspecified Effects of External Causes	29,795	29,270	1.79	1.69E-60	137.63

**Table 2:** Top 20 most enriched codes for Spring. The top 20 most enriched codes for Spring. Enriched codes include allergies, sprains and strains, and sports related injury. We report by percent increase as well as -log(p). We compare the number of codes found in Spring to the average code counts of the other three seasons

ICD 9 Code	Descriptions	Spring Code Counts	Average Count in Other Seasons	Percent Increase	*P* Value	Scores
840–848	Sprains and Strains of Joints and Adjacent Muscles	138,376	132,163.33	4.7	1.04E-66	151.93
995.3	Allergy, Unspecified	6304	5087	23.92	5.55E-61	138.74
990–995	Other and Unspecified Effects of External Causes	31,010	28,865	7.43	3.84E-36	81.55
905–909	Late Effects of Injuries, Poisonings, Toxic Effects, and Other External Causes	50,277	47,594	5.64	9.62E-35	78.33
995	Certain Adverse Effects not Elsewhere Classified	27,996	26,012	7.63	2.36E-34	77.43
980.9	Toxic Effect of Unspecified Alcohol	328	167.67	95.62	7.71E-28	62.43
844	Sprains and Strains of Knee and leg	18,141	16,806.33	7.94	1.71E-24	54.73
908.6	Late Effect of Certain Complications of Trauma	648	431	50.35	2.05E-22	49.94
E917.0	Striking Against or Struck Accidentally by Objects or Persons in Sports	2471	2020.33	22.31	3.06E-22	49.54
842	Sprains and Strains of Wrist and Hand	12,683	11,674.33	8.64	2.29E-20	45.22
848	Other and Ill-defined Sprains and Strains	16,380	15,278.67	7.21	8.61E-19	41.60
854	Intracranial Injury of Other and Unspecified Nature	17,691	16,558	6.84	2.01E-18	40.75
854	Without Mention of Open Intracranial Wound	17,515	16,401.67	6.79	5.34E-18	39.77
905	Late Effects of Musculoskeletal and Connective Tissue Injuries	23,970	22,703.33	5.58	4.87E-17	37.56
905.4	Late Effect of Fracture of Lower Extremities	9711	8915	8.93	7.21E-17	37.17
842.12	Sprain of Metacarpophalangeal (joint) of Hand	1659	1344.33	23.41	1.05E-16	36.79
842.1	Hand	5902	5296.67	11.43	2.50E-16	35.93
919.9	Other and Unspecified Superficial Injury of Other, Multiple, and Unspecified Sites, Infected	78	27.33	185.4	2.06E-15	33.82
854	Intracranial Injury of Other and Unspecified Nature Without Mention of Open Intracranial Wound, Unspecified State of Consciousness	13,228	12,381.67	6.84	3.94E-14	30.86
996	Complications Peculiar to Certain Specified Procedures	90,209	88,032.67	2.47	9.98E-14	29.94

**Table 3:** Top 20 most enriched codes for Summer. The top 20 most enriched codes for Summer. Enriched codes include accidents related to outdoor activities in warm weather such as bites and stings from bugs, burns, firework accidents, bicycle accidents, and water transport accidents. We report by percent increase as well as -log(p). We compare the number of codes found in Summer to the average code counts of the other three seasons

ICD 9 Code	Descriptions	Summer Code Counts	Average Count in Other Seasons	Percent Increase	*P* Value	Scores
E826-E829	Other Road Vehicle Accidents	5872	3166	85.47	5.54E-314	721.3
919	Superficial Injury of Other Multiple and Unspecified Sites	7846	4621	69.79	2.28E-301	692.25
E923.0	Accident Caused by Fireworks	480	44	990.91	2.97E-296	680.48
910–919	Superficial Injury	30,366	22,597.33	34.38	1.10E-290	667.65
919.4	Insect Bite, Nonvenomous, of Other, Multiple, and Unspecified Sites, Without Mention of Infection	2483	1067.33	132.64	7.41E-251	575.95
E826.1	Pedal Cycle Accident Injuring Pedal Cyclist	3933	2021.33	94.57	3.24E-246	565.26
997.91	Complications Affecting Other Specified Body Systems, Hypertension	1040	297.67	249.38	5.61E-220	504.84
940–949	Burns	45,311	36,094.67	25.53	3.83E-209	479.9
989.5	Toxic Effect of Venom	3019	1535.67	96.59	1.57E-195	448.56
E905.3	Sting of Hornets, Wasps, and Bees Causing Poisoning and Toxic Reactions	759	188	303.72	4.56E-195	447.49
800–829	Fractures	264,689	231,748.33	14.21	3.25E-171	392.56
E905	Venomous Animals and Plants as the Cause of Poisoning and Toxic Reactions	1006	350	187.43	1.06E-156	359.14
E830-E838	Water Transport Accidents	629	163	285.89	4.56E-153	350.78
989	Toxic Effect of Other Substances, Chiefly Nonmedicinal as to Source	3464	2016	71.83	8.47E-141	322.53
959.8	Other Specified Sites, Including Multiple Injury	17,695	13,440	31.66	1.21E-140	322.17
E923	Accident Caused by Explosive Material	927	335.67	176.16	1.66E-134	308.04
997.9	Complications Affecting Other Specified Body Systems	1262	535.67	135.59	3.50E-132	302.69
E826	Pedal Cycle Accident	5176	2631.67	96.68	0.00E+ 00	750
E900-E909	Accidents due to Environmental Factors	5367	3562.33	50.66	1.22E-116	266.9
E906.4	Bite of Nonvenomous Arthropod	1548	762	103.15	2.52E-111	254.66

**Table 4:** Top 20 most enriched codes for Fall. The top 20 most enriched codes for Fall. Enriched codes include motor vehicle accidents and sprains of neck. We report by percent increase as well as -log(p). We compare the number of codes found in Fall to the average code counts of the other three seasons

ICD 9 Code	Descriptions	Fall Code Counts	Average Count in Other Seasons	Percent Increase	*P* Value	Scores
E819.0	Motor Vehicle Traffic Accident of Unspecified Nature Injuring Driver of Motor Vehicle Other Than Motorcycle	1388	832.33	66.76	5.40E-70	159.49
E810-E819	Motor Vehicle Traffic Accidents	41,860	39,083.67	7.1	1.18E-48	110.36
E819	Motor Vehicle Traffic Accident of Unspecified Nature	17,872	16,481	8.44	5.12E-29	65.14
E819.1	Motor Vehicle Traffic Accident of Unspecified Nature Injuring Passenger in Motor Vehicle	777	518.33	49.9	1.53E-26	59.44
825	Fracture of one or More Tarsal and Metatarsal Bones	17,527	16,296	7.55	1.39E-23	52.63
900.9	Injury to Unspecified Blood Vessel of Head and Neck	557	366	52.19	8.43E-21	46.22
847	Sprain of Neck	18,864	17,707	6.53	8.62E-20	43.90
825	Fracture of Calcaneus, Closed	6243	5581.67	11.85	2.96E-19	42.66
E863.1	Accidental Poisoning by Insecticides of Organophosphorus Compounds	17	0.67	2437.31	1.43E-18	41.09
E980.9	Poisoning by Other and Unspecified Solid and Liquid Substances, Undetermined Whether Accidentally or Purposely Inflicted	565	383.67	47.26	2.74E-18	40.44
E949.6	Other and Unspecified Viral and Rickettsial Vaccines Causing Adverse Effects in Therapeutic use	37	6.33	484.52	6.33E-17	37.30
836	Dislocation of Knee	8608	7889.33	9.11	9.74E-17	36.87
999.9	Other and Unspecified Complications of Medical Care	1538	1241.33	23.9	1.54E-16	36.41
830–839	Dislocation	21,369	20,276.33	5.39	3.83E-16	35.50
E912	Inhalation and Ingestion of Other Object Causing Obstruction of Respiratory Tract or Suffocation	219	121.33	80.5	1.02E-15	34.52
E812	Other Motor Vehicle Traffic Accident Involving Collision With Motor Vehicle	13,813	12,965.67	6.54	6.65E-15	32.64
850.9	Concussion, Unspecified	2125	1793	18.52	7.41E-15	32.54
E812.0	Other Motor Vehicle Traffic Accident Involving Collision With Motor Vehicle Injuring Driver of Motor Vehicle Other Than Motorcycle	8037	7412	8.43	7.01E-14	30.29
E849.5	Street and Highway Accidents	7876	7266.67	8.39	1.66E-13	29.43
E881	Fall on or From Ladders or Scaffolding	1663	1386.33	19.96	1.97E-13	29.25

### Statistical enrichment for days of the year

**Fig. 1:**
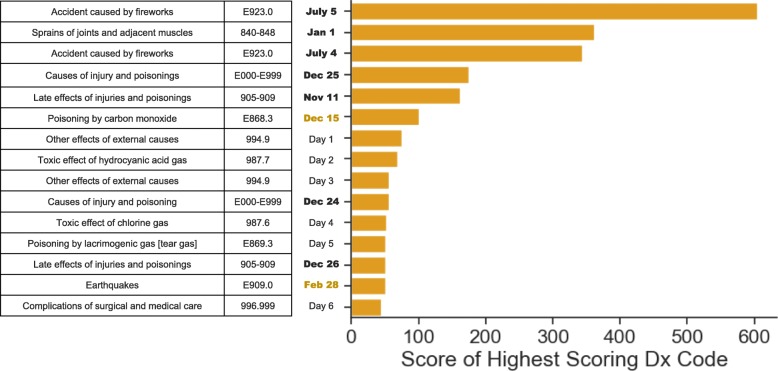
The top 15 highest scoring days of the year. The top 15 days with the highest scoring diagnosis codes. Each of the codes in the table are the most enriched codes on each of the days in the date column. The black bolded dates are either holidays or are dates that surround a holiday. The orange bolded dates are associated with known rare events that clearly explain the enrichment of their codes, namely the Nisqually Earthquake on Feb 28, 2001 and the Hanukkah Eve Windstorm on Dec 15, 2006. The other dates have unusual patterns of enriched codes such as chlorine gas poisoning and tear gas poisoning, but we could not find a readily available explanation to confirm some holiday, environmental, or social event on these days. Since these events appear to have happened on a single day in a single year and look to be associated with specific events, we have masked the dates due to the unknown specificity of these events and potential for identification of individuals involved in these events

To complement our seasonal analyses, we explored enrichment of diagnosis codes for all 365 days of the year. Each date that had a code scored below the Bonferroni threshold was flagged as having possible significance. We detected 100 days that had at least one code flagged as enriched. We generated an enrichment score for each of the dates by calculating the -log(*p*-value) of the lowest *p*-value for the date. The top 15 dates with the highest scoring codes are shown (Fig. 1). The days in which enrichment of many codes is common are a mixture of holidays and one time events. For example, there was enrichment of codes related to fights, firework accidents, and alcohol poisoning on January 1st (Table 5). Analogously, there was a large increase in the number of firework related accidents and burns on the 4th and 5th of July as well as an increase in the number of off-road vehicle accidents and poisoning by alcohol (Tables 6 and 7). We also observe an increase in alcohol poisoning, vehicle accidents, and an increase in possible self-harm on Christmas Eve (Table 8). For Tables 5, 6, 7 and 8, we limit the reporting of codes to those that had more than 30 appearances over the 24 years of data. This reduces false positives arising from extremely rare codes that appeared during the baseline period. We also report by percent increase rather than -log(p) for better interpretability.

**Table 5:** Top 10 most enriched codes for January 1st. The top 10 most enriched codes for January 1st. As expected for New Year’s Day, the most enriched codes were related to firework accidents, alcohol, and assaults. To reduce the false positive rate of the code enrichment from extremely rare codes that appeared during the baseline period, the enriched codes were only counted if they appeared more than 10 times over the 24 year period. We also report by percent increase rather than -log(p) for better interpretability

ICD 9 Code	January 1st Average Code Count	Daily Average Code Count	% Increase	Description
E923.0	1.74	0.07	2469.74	Accident caused by fireworks
E923	2.39	0.22	981.77	Accident caused by explosive material
E965	1.39	0.42	235.15	Assault by firearms and explosives
854.06	1.57	0.49	217.75	Intracranial injury with loss of consciousness of unspecified duration
E922.9	1.52	0.54	180.57	Accident caused by unspecified firearm missile
E922	1.87	0.69	171.55	Accident caused by firearm and air gun missile
E860	5.39	2.01	168.36	Accidental poisoning by alcohol, not elsewhere classified
E860-E869	5.96	2.23	167.25	Accidental Poisoning By Other Substance
E860.0	5.17	1.95	165.22	Accidental poisoning by alcoholic beverages
980.8	1.57	0.61	154.88	Toxic effect of other specified alcohols

**Table 6:** Top 10 enriched codes for July 4th. The top 10 most enriched codes for July 4th. As expected for Independence Day, the most enriched codes were related to firework accidents, burns, and alcohol poisoning. To reduce the false positive rate of the code enrichment from extremely rare codes that appeared during the baseline period, the enriched codes were only counted if they appeared more than 10 times over the 24 year period. We also report by percent increase rather than -log(p) for better interpretability

ICD 9 Code	July 4th Average Code Count	Daily Average Code Count	% Increase	Description
E923.0	4.26	0.06	6913.3	Accident caused by fireworks
E923	4.91	0.21	2194.4	Accident caused by explosive material
E820-E825	1.70	0.69	147.0	Motor Vehicle Non-traffic Accidents
980.8	1.43	0.61	133.5	Toxic effect of other specified alcohols
948.00	2.87	1.54	85.8	Burn involving less than 10% of body surface with third degree burn
948.0	2.87	1.56	83.8	Burn involving less than 10% of body surface
948	4.09	2.24	82.5	Burns classified according to extent of body surface involved
E819.2	3.00	1.70	76.2	Motor vehicle traffic accident of unspecified nature injuring motorcyclist
851	2.09	1.22	71.1	Cerebral laceration and contusion
851.8	1.35	0.79	69.9	Other and unspecified cerebral laceration and contusion, without mention of open intracranial wound

**Table 7:** Top 10 enriched codes for July 5th. The top 10 most enriched codes for July 5th. As expected for the day after Independence Day, the most enriched codes were related to firework accidents and burns as the injured persons from July 4th continue to appear in the hospital. To reduce the false positive rate of the code enrichment from extremely rare codes that appeared during the baseline period, the enriched codes were only counted if they appeared more than 10 times over the 24 year period. We also report by percent increase rather than -log(p) for better interpretability

ICD 9 Code	July 5th Average Code Count	Daily Average Code Count	% Increase	Description
E923.0	7.43	0.05	14,186.38	Accident caused by fireworks
E923	8.65	0.20	4144.02	Accident caused by explosive material
940	1.35	0.31	330.34	Burn confined to eye and adnexa
944.2	1.61	0.41	288.29	Blisters, epidermal loss [second degree] of hand, unspecified site
948.00	5.87	1.54	282.12	Burn involving less than 10% of body surface with third degree burn
948.0	5.87	1.55	278.04	Burn [any degree] involving less than 10% of body surface
948	7.48	2.23	235.42	Burns classified according to extent of body surface involved
944.2	4.57	1.40	225.29	Blisters, epidermal loss [second degree]
943.2	2.70	0.83	223.88	Blisters, epidermal loss [second degree]
941.2	2.74	0.86	218.77	Blisters, epidermal loss [second degree]
921.3	1.35	0.48	182.44	Contusion of eyeball

**Table 8:** Top 10 enriched codes for December 24th. The top 10 most enriched codes for December 24th. The most enriched codes were related to alcohol poisoning, injury to spleen, and injury undetermined inflicted. To reduce the false positive rate of the code enrichment from extremely rare codes that appeared during the baseline period, the enriched codes were only counted if they appeared more than 10 times over the 24 year period. We also report by percent increase rather than -log(p) for better interpretability

ICD 9 Code	December 24th Average Code Count	Daily Average Code Count	% Increase	Description
865.0	1.65	0.93	77.16	Injury to Spleen without mention of open wound into cavity
980.0	4.39	2.56	71.76	Toxic effect of ethyl alcohol
865	1.65	1.01	64.33	Injury to spleen
E980-E989	2.96	1.87	57.91	Injury Undetermined Whether Accidentally Or Purposely Inflicted
E980	1.83	1.17	55.93	Poisoning by solid or liquid substances
980	4.57	3.29	38.70	Toxic effect of alcohol
E812.1	1.96	1.46	34.11	Other motor vehicle traffic accident involving collision with motor vehicle injuring passenger in motor vehicle other than motorcycle
E816	2.22	1.73	28.51	Motor vehicle traffic accident due to loss of control
E849.9	2.65	2.21	19.85	Accidents occurring in unspecified place
E819.9	5.17	4.61	12.31	Motor vehicle traffic accident of unspecified nature

### Rare events as case studies

We detected enrichment of unusual codes on multiple days that did not seem linked to their respective day by either holiday or seasonal event. Upon further evaluation, we inferred that we had detected past environmental events that showed up as single day enrichments. Feb 28, Dec 15, May 31, and Nov 8 were four of the days in the top 15 highest scoring days that followed this pattern (Fig. 1). Because these enriched days fell in single years, we were able to search for news stories published on or immediately after these days to see if we could find the cause of the increase in these unusual codes.

#### Nisqually earthquake

In our analysis, February 28th was shown to have an increase in earthquake related accidents, ICD-9-CM code E909.0. On February 28, 2001, there was a magnitude 6.8 earthquake centered in Western Washington [24, 25]. All the earthquake codes found on February 28th in our dataset were from 2001, consistent with there being very few earthquake related accidents in the EHR except during the major earthquake.

#### Hanukkah eve windstorm

Our event detection method also discovered a significant increase on December 15 of the ICD-9-CM code E868.3 (accidental poisoning by carbon monoxide from incomplete combustion of other domestic fuels). Nearly all the codes were found to have been coded in 2006. The Hanukkah Eve windstorm of Dec 15, 2006 led to widespread and lengthy power outages. In the aftermath, there were news stories about the increase in carbon monoxide poisonings due to people barbecuing and running generators in their homes without ventilation [26, 27]. Indeed, public health authorities responded with concerns that the dangers of carbon monoxide poisoning were not widely understood in select communities [28].

#### Industrial accidents

We detected two other single day enrichments: May 31 with an enrichment of E891.3 (Burning caused by conflagration) and Nov 8 with an enrichment of 987.6 (Toxic effect of chlorine gas). We were able to link these two enrichments to the May 31, 2004 monorail fire in Seattle [29] and the November 8, 1994 chlorine spill and fire at the Coastal Dock in Ballard, WA [30].

## Discussion

We explored the value of UW Medicine electronic health record data for detecting public health-related environmental and seasonal causes of traumatic injury. Our analysis finds that tests for seasonal and daily enrichment of the frequency of emergency room visits for trauma detects expected events, including both seasonal trends such as winter sports-related injuries, day-specific events such as July 4th burns, and rare events such as the Nisqually earthquake.

### Interesting anomalies

#### Non-enriched holidays

While most of our results confirmed expected seasonal and date-specific trends, we were surprised not to find enrichment of alcohol related injuries on St. Patrick’s Day or the day following, given that St. Patrick’s Day is associated with increased alcohol consumption [31, 32]. This may indicate the effectiveness of extra police patrols deployed for that day. This could also be a false negative due to the conservative nature of Bonferroni corrections.

Prior studies have examined date-related events in relation to traumatic injury. One study found that on April 20th, a date associated with celebrating marijuana consumption, there was an increase in the number of car accidents [33]. While we did not observe a statistical enrichment in car accidents, our method did identify a statistical enrichment in burns (940–949), another potential consequence of marijuana use [34]. Future work could analyze clinical notes which might allow us to identify if this enrichment is attributable to elevated marijuana use.

#### Enrichment of post-surgical complications in winter

**Fig. 2:**
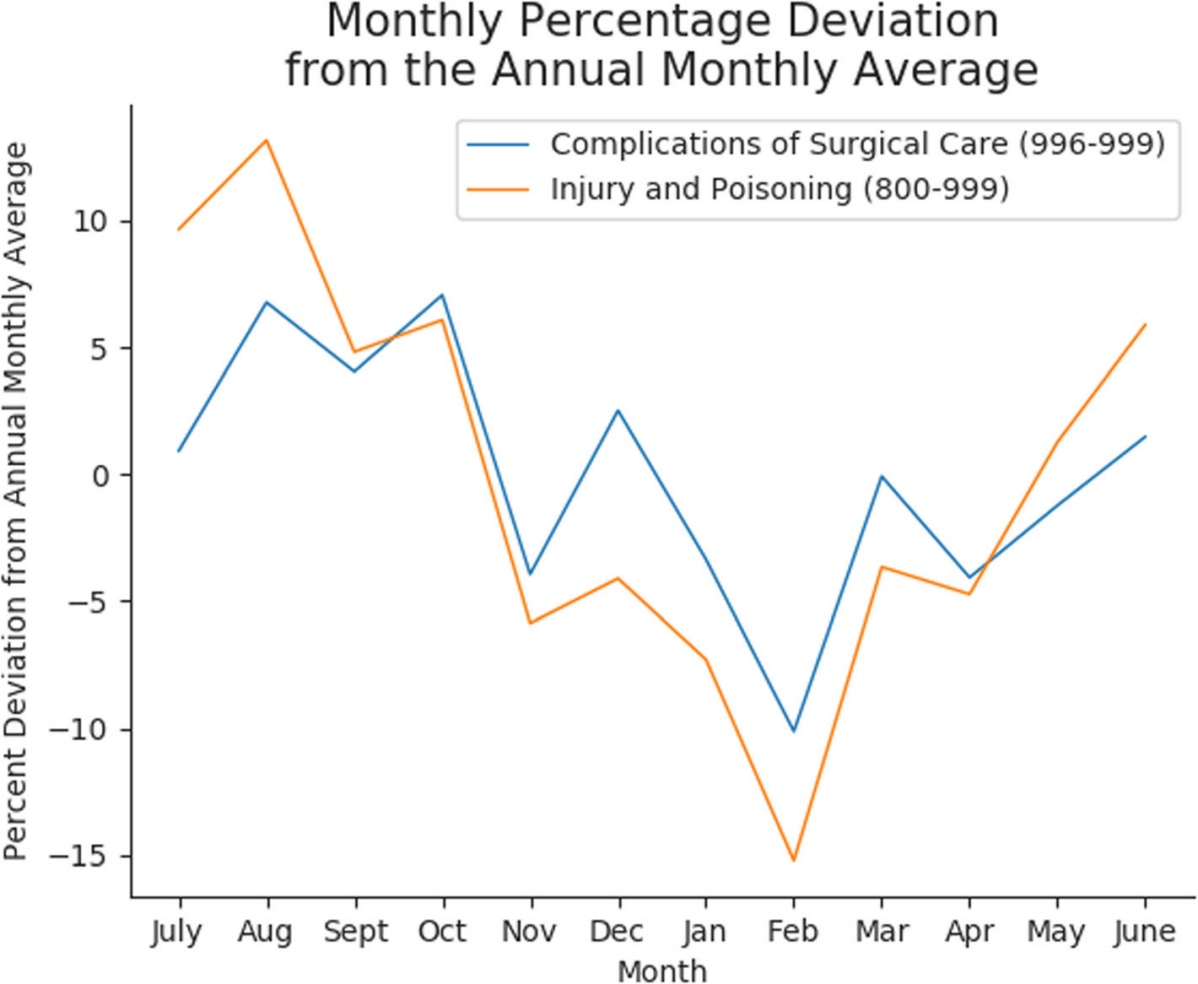
Comparison of the code count trend differences between 996 and 999 and 800–999 The percent deviation from the annual monthly average code count for both the Complications of Surgical Care (996–999) diagnosis family and the broad category of Injury and Poisoning (800–999). By calculating the average monthly code count for each family and the percent deviation per month from that expected average, we see that both code families follow a similar seasonal pattern of increase in the summer and decrease in the winter in terms of raw code count. While they follow the same pattern, Complications of Surgical Care doesn’t decrease as much in the winter, and actually has a spike in December, which is why our method picks up this diagnosis family as enriched in the winter. Since the number of trauma visits is used to establish a baseline expected rate of each code count, our method is detecting relative enrichment and not absolute enrichment

We also saw unexpected trends in post-surgical complications, with those terms being enriched in the winter months at the very end and beginning of the year. One hypothesis is that there is a relative increase in the number of surgeries in November and December as people schedule elective surgeries before insurance deductibles reset in the new year. An alternate hypothesis is that people defer reporting minor surgical complications until after the end-of-year holidays. We were unable to explore these hypotheses for this study because our data was limited to visits including trauma codes and did not include surgical appointments. It is also important to note that we saw a relative increase in the number of surgical complications due to lower numbers of trauma visits in the winter, and not necessarily an absolute increase in the number of post-surgical complications (Fig. 2). Since codes related to post-surgical complications are less specific and are more likely to appear during trauma visits than other codes discussed thus far, the effect of this “lowered baseline” is particularly noticeable.

#### Unlinked events

There were multiple dates that had significant enrichment of codes on a date where nearly all the codes came from one year. For instance, there were a large number of visits with the code 994.9 (other effect of external causes) on one of the masked days. This code is too vague to understand the common injuries of patients and, at the time of this study, we did not have access to de-identified clinical notes from which to elicit the causes of these injuries. There was also no readily available source of news that we found to corroborate a large number of people being injured by any social or environmental event. We were not able to discern whether these dates were false positives, whether the codes were entered incorrectly, or whether there was a common event that caused these injuries. In this paper, we have masked the specific dates of these unlinked days to protect against the potential de-identification of patients since the circumstances surrounding these injuries are unknown.

### Study strengths and limitations

Our study has several notable strengths. First, the UW Medicine system has used EHRs for a long time, affording us access to over 20 years of clinical data from a large urban health care system. Second, UW Medicine’s location in Western Washington lends itself to year-round yet season-specific outdoor activities whose resulting injuries show up as specific trauma codes, including snow sports in the winter and boating in the summer. This access increased our ability to detect seasonal trauma trends.

However, our study also has limitations. First, as with any study of electronic health records, we cannot rule out biases due to site-specific coding practices or changes in practitioner knowledge of the health record system. However, we have no reason to believe errors caused by these issues would vary by season or day. Second, the UWMC is mainly a referral institution, such that many patients visit the system only for specialty services. We also know that only around 31% of all patients visiting the UW medical system will have their next visit at a UW clinic [35]. This is mitigated in our study by the fact that we only considered trauma-related diagnosis codes and that UW Medicine is the only Level I trauma center in Washington, Alaska, Montana and Idaho. The impact of this known bias decreases since our study looks at individual admissions and does not require a full picture of each patient odyssey. The results of our study are not reliant on continuity of care. Nevertheless, further validation studies are needed to evaluate the representation of the UWMC data in the Seattle Region. Another future solution would be to run our method at more sites across Washington, feeding the live statistics into an aggregation mechanism for a more robust population view.

### Using electronic health Records for Event Detection

Our method could be used in a live surveillance situation by alerting authorities and doctors when an unusual increase of cases with a particular diagnosis code show up across multiple hospitals with linked EHR systems. It could spark an investigation into what is causing the sudden increase but also could initiate public health policy development that previously would take longer to assess and carry out. While our method focused on traumatic injury, it could easily be expanded to include surveillance of all diagnosis codes. A limitation of using billing codes for surveillance is the delay that occurs between patient care and the billing process. While this delay is shorter than periodically collecting all the latest billing codes, a true real-time surveillance system isn’t possible. A possible next step would be to train an NLP classifier based on the clinical note texts from each visit to “predict” the diagnosis codes that will be associated with a visit. While not a trivial pursuit, this would enable a near real-time surveillance system. Aside from predicting diagnosis codes, incorporating clinical notes into the method could more accurately cluster events and better inform detected trends. Natural language processing techniques could be used to find “enriched” keywords on the detected days to add context to the detected events in a data driven automated manner.

## Conclusion

In conclusion, electronic health record data hold considerable potential for public health surveillance. We explored the potential to leverage UW Medicine’s enterprise data warehouse to detect seasonal, holiday, and rare events using diagnosis codes for injuries and poisonings. Our method detected many of the trends for seasons and specific dates we expected, while identifying several intriguing new enrichments. Future research should focus on improving our trend and event detection method to differentiate between one-time effects like the Nisqually earthquake, and repeat events like Independence Day. Incorporating clinical notes into a detection method could more accurately cluster events and better inform detected trends. Expanding the method to all diagnosis codes could detect new non-trauma related events. Our findings add to the growing body of literature showing that electronic health records hold considerable potential as generalizable population health surveillance platforms.

## Supplementary information

**Additional file 1.** Data Processing Methods. Description of data processing methods This file details the methods and rationale used to clean and process the raw clinical data into study ready data. The description includes the mapping process for converting ICD-10-CM diagnosis codes to ICD-9-CM, the data sources for this process, and the rationale for the decisions made.

## Data Availability

The datasets generated and analyzed during the current study are not publically available.

## Abbreviations

BRFSS: Behavioral Risk Factor Surveillance System
EDW: Enterprise Data Warehouse
EHR: Electronic Health Record
ICD-10-CM: International Classification of Diseases, Tenth Revision, Clinical Modification
ICD-9-CM: International Classification of Diseases, Ninth Revision, Clinical Modification
UWMC: University of Washington Medical Center
