# Supplementary material for: Evaluation of the secondary use of electronic health records to detect seasonal, holiday-related, and rare events related to traumatic injury and poisoning
Source: BMC Public Health. 2020 Jan 13;20:46. doi: 10.1186/s12889-020-8153-7 (PMC6958939; doi:10.1186/s12889-020-8153-7)
Supplement: Supplementary file 1 — Additional file 1. Data Processing Methods. Description of data processing methods This file details the methods and rationale used to clean and process the raw clinical data into study ready data. The description includes the mapping process for converting ICD-10-CM diagnosis codes to ICD-9-CM, the data sources for this process, and the rationale for the decisions made. [file 12889_2020_8153_MOESM1_ESM.docx]

**Supplementary Material**

**Data Cleaning**

**Diagnosis Coding Method**

Since there are only two versions of ICD used in this dataset, all values were mapped to either ICD-9-CM or ICD-10-CM.

1. Convert all characters to uppercase
2. Remove all whitespace
3. Map all terms to standard format using the Dx code method mapping dictionary

{

'I9':'ICD-9-CM',

'I10':'ICD-10-CM',

'O9':'ICD-9-CM',

'ICD10':'ICD-10-CM',

'ICD9':'ICD-9-CM',

'ICD-10-CA':'ICD-10-CM'

}

**Admit Datetime**

All the dates were consistent in their format. (Year-Month-Day)

**Diagnosis Codes**

ICD-9-CM was the diagnosis coding standard until 2015, when the University of Washington transitioned to using ICD-10-CM. Since the vast majority of the diagnosis codes are already in ICD-9-CM, we chose to convert all the ICD-10-CM codes to their nearest equivalent code/codes in ICD-9-CM. We used the Centers for Medicare and Medicaid Services General Equivalence Mappings (CMS GEM) to convert ICD-10-CM to ICD-9-CM (<https://www.cms.gov/Medicare/Coding/ICD10/2017-ICD-10-CM-and-GEMs.html>).

One of the issues with converting ICD-10-CM to ICD-9-CM is the “one to many” or “many to one” conundrum. A single ICD-10-CM code can be mapped to one ICD-9-CM code, to a couple combined codes, or to multiple sets of combined codes that can be chosen depending on the clinical situation. Since we are dealing with large sets of data and since all of the combined codes are related in some way to the original ICD-10-CM code, we used all codes that could be mapped to the original ICD-10-CM code, even if the codes are meant to be separated into groups. In this way, we can create a general equivalency bag of terms that might add small amounts of information but would ensure the highest information retention.

**Diagnosis Code Hierarchy Structure and Sources**

The ICD-9-CM concept hierarchy is a non-binary tree structure with a max depth of 7. The ICD-9-CM hierarchy was put into an easily computable JSON format by Dr. Eugene Wu (<http://www.eugenewu.net/>), a researcher in the computer science department at Columbia University. We retrieved the JSON file at his github <https://github.com/sirrice/icd9>. Since there were entire families missing from this hierarchy, the missing information was manually retrieved from [www.icd9data.com](http://www.icd9data.com), a website owned and operated by Alkaline Software. We treated the icd9data website as the ground truth for our hierarchy. If Wu’s hierarchy deviated from the icd9data hierarchy, we used the icd9data version of the code structure. In this way, we have developed a complete json formatted version of the entire ICD-9-CM concept hierarchy.

Since we will not be using ICD-10-CM, describing the structure is not as important, however, there are some caveats that need to be addressed before converting from ICD-10-CM to ICD-9-CM. The CMS GEM files we used were for the 2017 version of ICD-10-CM, therefore, if a code appears in our EHR data but was coded prior to 2017, there is a chance that it may not appear in the 2017 CMS GEM file. Before we could run the code through the GEM file, we needed to first cross reference the code against the 2017 ICD-10-CM Conversion Table to check which codes changed from the previous year and to change the 2016 version to the 2017 version.

The functions and JSON files used for the conversion and analysis of the diagnosis codes are available in a custom built tool on the Mooney Lab github: <https://github.com/UWMooneyLab/DxCodeHandler>

**Diagnosis Code Column Processing**

1. Convert all values to uppercase
2. Strip off all whitespace
3. If Diagnosis Coding Method is ICD-9-CM
   1. Cross reference against the developed JSON ICD-9-CM hierarchy to ensure the code exists.
   2. If the code does not exist, remove the right-most character from the code. Often, non-existent billable leaf codes such as 800.07 will be used, but the existing code 800.0 can capture the higher concept, thus we strip down the code until it exists in the hierarchy.
4. If Diagnosis Coding Method is ICD-10-CM
   1. Cross reference the code against the 2017 conversion table and convert to the 2017 equivalent ICD-10-CM code if possible.
   2. Using the CMS GEM, we try to find the ICD-10-CM code in the GEM file and convert the existing codes to a bag of concept ICD-9-CM code list.
   3. If the ICD-10-CM code does not exist in the CMS GEM file, we return NoDx and the visit information is removed from our dataset.
   4. Remove all rows with diagnosis codes where the Admit Datetime is null or blank.
